# Students’ Personality Contributes More to Academic Performance than Well-Being and Learning Approach—Implications for Sustainable Development and Education

**DOI:** 10.3390/ejihpe10040079

**Published:** 2020-12-06

**Authors:** Paulo Moreira, Susana Pedras, Paula Pombo

**Affiliations:** 1 Instituto de Psicologia e de Ciências da Educação [Institute of Psychology and Education], Universidade Lusíada-Norte, 4369-006 Porto, Portugal; pmpombo@por.ulusiada.pt; 2 Centro de Investigação em Psicologia para o Desenvolvimento (CIPD) [The Psychology for Positive Development Research Center], Universidade Lusíada-Norte, 4369-006 Porto, Portugal; susanapedras@gmail.com

**Keywords:** academic performance, personality, learning approach, affective well-being, non-affective well-being

## Abstract

The present study aimed to describe the predictive role of personality dimensions, learning approaches, and well-being in the academic performance of students. In total, 602 students participated in this cross-sectional study and completed a set of questionnaires assessing personality, learning approach, and well-being. Two indexes were calculated to assess affective and non-affective well-being. The results partially support the hypotheses formulated. Results revealed that personality temperament and character dimensions, deep learning approach, and affective well-being were significant predictors of academic performance. A deep approach to learning was a full and partial mediator of the relationship between personality and academic performance. The results improve the understanding of the differential contribution of personality, type of learning approach, and type of well-being to academic performance. Comprehending that personality is the strongest predictor of academic performance, after controlling the type of learning approach and the type of well-being, informs school policies and decision-makers that it is essential to encourage personality development in adolescents to improve academic performance. These results also have implications for educational policies and practices at various levels, including an emphasis on the role of well-being as an educational asset. Understanding the links between personality, well-being, and education is essential to conceptualize education as a vital societal resource for facing current and future challenges, such as sustainable development.

## 1. Introduction

Academic performance results from interactions between several factors. In addition to the classic variables, such as intelligence (through capabilities) or socioeconomic level (through stimuli and opportunities), personality is a well-known predictor of academic performance. The role of personality of high school and university students for their academic performance is well-known [1,2,3,4,5] but its relationship with well-being is relatively unclear. According to Poropat [3], the relationship between personality and academic performance changes, especially between 11 to 16 years old, and well-being seems to decrease along with adolescence [6,7]. In addition, contemporary perspectives emphasize the importance of other variables, such as the type of approach to learning preferred by students, in understanding academic processes and results, as well as personality and well-being.

Academic performance is usually measured by the final grade obtained in the course, which is one of the most studied indicators of academic success. In view of the growing pressures for academic success, academic performance is of great importance for students, teachers, and the national education system responsible for the formulation and implementation of educational policies. Thus, there are theoretical reasons to believe that relatively stable individual traits (personality), a school and study-based variable (learning approach), and a more volatile individual variable (well-being), will be interrelated. Therefore, knowledge of these relationships will be useful not only to inform school policymakers, but also to inform educators and parents about which adolescents need more support, and in what areas, to achieve greater academic success.

Eccles and colleagues developed the Value-Expectancy Model, which is a relevant model for understanding achievement motivation, including academic achievement and academic performance. This model has been recently updated as a developmental, social cognitive and sociocultural perspective on motivation [8]. According to this model, students’ academic performance is strongly influenced by student achievement motivations, which are shaped by individual characteristics. Amongst the individual characteristics involved in the construction of achievement motivation, temperament and personality play important roles in students’ preferences for certain learning approaches. Although temperament and personality are relatively stable and dispositional dimensions, students’ motivations, including achievement motivations and academic performance, tend to be influenced by students’ emotional states, including well-being (both affective and non-affective well-being). Although research describing the influences of each one of these variables on academic performance is abundant, studies on the interaction between personality (including temperament), learning approaches, and well-being in predicting academic performance are scarce, and is the reason why this is the main objective of the present study.

### 1.1. Temperament and Character Dimensions of Personality

Sociocognitive models of personality are seen as an effort to model the structure of intra-individual personality and an attempt to explain personality by formulating conceptual models of the mental architecture underlying human experience and action patterns. These models consider the processes of knowledge construction as central to the human being and, therefore, must be central to the theoretical models of personality [9,10,11].

Cloninger and colleagues developed the Psychobiological Model of Personality that conceptualizes the personality as an organization of dynamic and nonlinear psychobiological processes [12], i.e., “the way a person learns to adapt to experience, or, more specifically, as the dynamic organization within the individual of the psychobiological systems by which a person both shapes and adapts uniquely to an ever-changing internal and external environment” ([13], p. 1). Thus, this model integrates genetic, neuro, and psychobiological aspects of the human personality into two dimensions: temperament and character. Temperament refers to innate individual differences, in associative responses to basic emotional stimuli that shape emotional habits and responses, measurable at the beginning of development, and reflected in brain structures and functions [12]. In addition, temperament refers to individual differences in associative conditioning and related human brain circuits [13,14]. In turn, character pertains to the self-regulating aspects of the personality, that is, the way a person shapes and adapts responses to external and internal conditions [12], including the executive, legislative, and judicial functions, necessary for the mental self-government and self-actualization of identity [15].

Four dimensions of temperament capture these individual differences: novelty seeking, harm avoidance, reward dependence, and persistence. Each extreme of temperament has advantages and disadvantages depending on the situation. Novelty seeking and harm avoidance are responsible for activating and inhibiting behaviors [12]; that is, novelty seeking is the tendency to respond to new stimuli, while harm avoidance is the tendency to inhibit behavior in the presence of aversive stimuli. In addition, novelty seeking reflects individual differences in the brain’s behavioral activation system, which is crucial for learning and for regulating motor habits and skills [12]. In turn, harm avoidance has an inhibitory inclination. It reflects the activity of the punishment system, a threat-processing device that anticipates, detects, and responds with defensive actions to hazards or threats [15]. The other two dimensions of temperament are responsible for maintaining behavior: reward dependence and persistence. Reward dependence is the tendency to respond positively and maintain behavior in the presence of signs of reward and social approval. Persistence, in turn, represents the tendency to persevere in long-term goals and maintain the behavior despite the frustration, fatigue, and lack of reward [12].

The three dimensions of character are self-directedness, cooperativeness, and self-transcendence. Self-directedness refers to an individual’s willpower or ability to control, regulate, and adapt his behavior to achieve relevant personal goals and values [12]. It also represents the individual’s ability to control his conduct and guide him towards personal goals and objectives, using his resources appropriately [16]. Cooperativeness refers to the empathic ability to accept others and identify their emotions and, if necessary, to forget personal gratifications for the benefit of the social group. Cooperativeness is related to an individual’s tolerance and acceptance, his ability to be sensitive to external needs, his tendency to help and manifest pro-social values, and to establish interpersonal exchanges [16]. Self-transcendence refers to how well individuals identify themselves as an integral part of the universe as a whole and their experience of something superior that goes beyond ourselves [12]. Individuals with high self-transcendence are prone to creativity, magical thoughts and religiosity.

Cloninger’s Psychobiological Personality Model was used in this study and not the Big Five Personality Model for three reasons. First, the Big Five Personality Model is a lexical model, the structure of which derives from linear data reduction, which does not provide a comprehensive conceptual explanation of how personality works [17,18]. For example, the Big Five Model does not distinguish qualitatively different processes, such as the emotional and cognitive components of the personality. Previous research has shown consistent and positive associations between neuroticism and the surface approach to learning, and between conscientiousness and the deep approach to learning [19,20]. Recent research has shown that neuroticism is a factor that comprises two qualitatively different psychobiological processes: high anxiety and low self-directedness. In turn, conscientiousness encompasses aspects of persistence and self-directedness [21,22]. In addition, in a recent study that examined the influences of Cloninger’s Psychobiological Personality Model on learning approaches, Moreira and colleagues [23] found that although students’ preferences for deep and surface learning approaches are best understood as integrated temperament-character profiles, temperament and character dimensions have independent significant effects on learning approaches. Finally, we chose to use Cloninger’s Psychobiological Personality Model, on the one hand because it conceptualizes temperament and character dimensions independently, and on the other hand because Eccles’ Expectation-Value Model, used in the framework of this study, explicitly refers to the emotional (temperament) and cognitive dimensions of the personality as distinct factors that exert an independent influence on performance motivation [8].

Adolescence is a period characterized by personality changes that influence developmental, emotional, social, and academic results. Although the personality is relatively stable, it is changeable and manifests itself differently at specific ages. Recently, Zohar et al. [24] found that temperament and character traits were only moderately stable from 12 to 16 years old. In particular, harm avoidance and persistence have decreased, while self-directedness and cooperativeness increased from 12 to 16 years old. The novelty seeking, reward dependence, and self-transcendence increased from 12 to 14 years and then decreased [24]. Therefore, during adolescence, personality dimensions can have substantially different influences, depending on the results we are evaluating.

### 1.2. Learning Approach

This construct refers to the relationship that students develop with learning tasks, a process that combines motivational guidance, and a type of learning strategy [25,26,27]. Thus, the learning approach refers to the understanding and meaning of the students’ learning experience, which is associated with personal (cognitive, affective, and interpersonal) and environmental factors (educational goals, content, methods, materials, resources) that influence and affect the academic processes and outcomes [28]. Knowing the type of approach students prefer and adopt, allows us to understand how students relate to learning tasks, in order to promote the understanding of individual variability at the study level [26]. Marton and Säljö [28,29] identified two contrasting approaches: a surface approach and a deep approach to learning [30,31,32,33,34,35].

The deep approach is characterized by the student’s underlying guiding intention to maximize intellectual understanding and extract meaning from the task, i.e., it presupposes the existence of intrinsic motivation. The student seeks to understand and establish relationships between concepts, generalize learning to new concepts, and different situations. Students who take this approach have an active interest in the themes and use logic to understand the concepts [25,26].

The surface approach is characterized by the existence of extrinsic task-oriented motivation and a superficial strategy. This approach is characterized by mechanical and reproductive learning, using the memorization of content, with low commitment and effort on the part of the student, with minimal time spent, but with anxiety to face demanding learning tasks. Surface motivation is considered instrumental, and the student’s goal is to learn the minimum necessary to fulfill what is required, pass the exam, and avoid failures [25,26].

Overall, existing studies suggest a significant and positive relationship between the deep learning approach and academic achievement [36,37,38]. Although there are several studies on the coexistence of the two learning approaches, as well as the prominence of each other, the results are inconsistent or have small effects and, therefore, cannot be generalized for all contexts [39,40,41]. This is mainly due to cultural, social, and contextual/learning environment factors.

Regarding personality and academic performance, based on the Big Five Model, studies show that agreeableness and openness to experience are positively associated with motivation for achievement, more effective involvement in educational experiences, and a deeper approach to learning [42,43]. Conscientiousness is associated with greater objective orientation [43] and extroversion with mastery, approximation, and performance objectives [32]. Neuroticism is associated with the avoidance of academic motivation (suggesting that students avoid aspects of academic life) and with a surface learning approach [27].

Recently, Moreira et al. [23], based on the Psychobiological Model of Personality, used a person-centered approach to assess the relationship between personality profiles and students’ preferred approach to learning. The authors found two profiles, one defined by less novelty seeking, greater reward dependence, and persistence that they labeled as “steady” profile, and the second was defined by greater novelty seeking, less reward dependence, and persistence, which was labeled as “disinhibit” profile. The results suggest that students with a “steady” temperament showed a preference for the deep approach to learning. Students with high character coherence also had this preference. A temperament profile-by-character profile interaction was crucial for understanding students’ preferred approach to learning, and implies that adaptive learning approaches result from an integration of the main learning and memory systems, as measured by the Junior Temperament and Character Inventory (JTCI).

### 1.3. Affective and Non-Affective Well-Being

The school is not just a place of excellence for learning. The school is also the place where adolescents can be happy and healthy, where they can make friends, develop social and emotional skills, and develop their personality. Thus, the school is a privileged place for the promotion of well-being, whether affective (associated with experiences of positive and negative situations and events) or non-affective (associated with the perception of social support, satisfaction, and quality of life) [44,45]. Affective well-being refers to the frequency and intensity of positive and negative emotions and mood. Non-affective or cognitive well-being refers to specific domains and global evaluations of life such as social support, quality of life and global life satisfaction. As a result, adolescent well-being is associated with several indicators of developmental trajectories [46], including school involvement [47,48,49,50] and academic achievement and performance [50,51]. Well-being is also a protective factor for negative health outcomes [52]. Adolescents with higher levels of well-being are more resilient [53,54], show less delinquency and aggressive behaviors, lower level of depression and anxiety symptoms, greater self-esteem, sense of effectiveness, and adaptation [53,54,55]. In addition, adolescents with high persistence and self-directedness showed higher well-being [56,57].

Thus, studies suggest that students who adopt a deep learning approach with mastery goals, greater involvement in self-regulated learning, and with the use of metacognitive skills, have a better academic performance. On the other hand, students who engage in academic tasks to demonstrate skills, reveal biased results of a negative pattern as they adopt surface learning strategies [58,59]. In addition, temperament and character dimensions are associated with learning, since learning is considered an organization of behavior as a result of individual experience [12]. In the study by Rosa and Moreira [60], the combination of certain personality dimensions (persistence and self-directedness) with certain learning approaches (surface and deep learning approach) explained 22% of the variance in academic performance. Interestingly, learning strategies also proved to be a significant mediator on the relationship between students’ interest in history and their achievement [61].

Therefore, this study aimed to know: (1) the dimensions of personality, (2) the type of learning approach, and (3) the type of well-being (affective and/or non-affective) contributing to academic performance, (4) which variable (personality, learning approach or well-being) contributes the most to academic performance, and, finally, (5) whether the type of learning approach plays a mediating role in the relationship between personality dimensions and academic performance.

Thus, considering the differential effects of personality, together with the role of the learning approach and the well-being of students, for academic performance, the following hypotheses were formulated:

**Hypothesis 1** **(H1).** *personality dimensions are expected to contribute more to academic performance, regardless of the learning approach chosen by students;*

**Hypothesis 2** **(H2).** *well-being is expected to contribute more to academic performance, regardless of the learning approach chosen by students;*

**Hypothesis 3** **(H3).** *the deep learning approach is expected to be a mediator in the relationship between the dimensions of personality and well-being.*

**Hypothesis 4** **(H4).** *more well-being (affective and non-affective), a deep approach to learning, and more persistence, self-directedness, and cooperativeness, are expected to contribute positively to academic performance.*

The results will allow to understand the role and contribution of these variables for students’ academic performance. These variables can be modified and promoted through emotional, social, and academic well-being programs implemented in the school context.

## 2. Method

### 2.1. Sample

The study included 602 high school students (10th to 12th grade) from five schools in the north of the country, 346 female students (57.5%), 256 male students (42.5%) aged 14 to 17 years old (M = 16.07, SD = 0.8). Most of the students were attending regular education in scientific-humanistic courses (*n* = 490, 81.4%), specifically in socio-economic sciences (*n* = 32, 6.5%), social and human sciences (*n* = 21, 4.9%), and science and technology (*n* = 434, 88.6%). The remaining sample (*n* = 112, 18.6%) were enrolled in vocational courses. The grade point averages (GPA) of the total sample (values for n = 593 students in the total sample) was 13.56 (SD = 1.96) on a scale of 0 to 20, ranging from 8 to 19 in this sample.

### 2.2. Procedure

Students were invited to participate voluntarily in this study and were recruited from five schools in Northern Portugal, according to the snowball technique for the selection of nonrandomized samples. All students who collaborated presented their parents’ written informed consent and were gathered in a 1 h group session to complete the questionnaires in the presence of a member of the research team. The research protocol (Reference CIPD_Academic performance_20080) was approved by the Ethics Council on Behavioral Research of the Universidade Lusíada-Norte, Porto, and by the Directors of the schools where the data collection took place.

### 2.3. Measures

Sociodemographic Questionnaire: The sociodemographic characteristics of adolescents, such as age, gender, and grade were collected.

Academic performance: Grade point average (GPA) was collected on a scale from 0 to 20.

Learning Process Inventory, LPI [31,62]. This questionnaire consists of 19 items that evaluate the Deep Approach to Learning and 14 items that evaluate the Surface Approach to Learning. The higher the result, the greater the student motivation and learning strategies in a given learning approach. The Portuguese version [31] has good psychometric characteristics with an alpha of 0.83 for both approaches. The Cronbach’s alpha in this study for the Deep scale was 0.95 and for the Surface scale, it was 0.91.

Junior Temperament and Character Inventory, JTCI [12,63]. It consists of 127 items that measure the seven major dimensions of the Psychobiological Model of Temperament and Character. The dimensions and alphas were as follows for the four JTCI Temperament dimensions: Novelty Seeking (NS): 0.61; Harm Avoidance (HA): 0.50; Reward Dependence (RD): 0.32; Persistence (PS): 0.38. The three dimensions of Character and the respective alphas were as follows: Self-directedness (SD): 0.77; Cooperativeness (CO): 0.83; and Self-Transcendence (ST): 0.72. The Portuguese version [64] has good psychometric characteristics above (0.60) except in the dimension reward dependence (0.57).

KIDSCREEN-10 [65]. This 10-item scale assesses the quality of life in children/adolescents and higher results indicate greater satisfaction with the quality of life. The Portuguese version [66] has good psychometric characteristics (0.78). In this study, Cronbach’s alpha was 0.78.

Brief Student Life Satisfaction Scale, BSLSS [67]. This 6-item scale assesses satisfaction with life and higher results indicate greater satisfaction with life. The original version of this scale has an alpha of 0.75. This scale has already been used in a study with a similar sample [68] and, in this study, Cronbach’s alpha was 0.84.

Satisfaction Scale with Social Support for Children and Adolescents, SSSS [69]. This scale includes 12 items and higher results indicate greater satisfaction with social support. Portuguese version [69] has good psychometric characteristics (0.84). In this study, Cronbach’s alpha was 0.70.

Positive Affect and Negative Affect Scale, PANAS [70,71]. This scale includes 10 items that evaluate positive affect (PA) and 10 items that evaluate negative affect (NA) and higher results indicate higher PA and NA. The Portuguese version [71] has good psychometric characteristics (PA 0.86; NA 0.89). Cronbach’s alphas in this study were as follows: 0.90 for PA and 0.92 for NA.

Composite Non-affective Index and Affective Index. We estimate the two indices as indicators of non-affective and affective well-being, respectively. We follow the suggestion of Cloninger and Zohar [44] and Josefsson et al. [45] for this estimate. The Non-Affective Index (non-affective well-being) refers to the average of satisfaction with social support, life satisfaction, and health-related quality of life. The Affective Index (affective well-being) was estimated by the positive affect score minus the negative affect score; it thus reflects the emotional tone of the individual’s experience: the salience of positive emotions (desirably present) and negative emotions (desirable absence). These indices were already used in similar samples [68].

### 2.4. Statistical Analysis

This is a cross-sectional study. The sample characteristics were analyzed using descriptive statistics. Pearson’s coefficients were calculated to examine the relationship between the study variables. The dependent variable (academic performance) was filled in by 593 students. Missing values were not replaced, taking into account the type of qualitative variable we are dealing with, so only 593 participants were included in the analysis and not 602. To assess the degree to which personality dimensions, type of approach to learning, affective and non-affective well-being differentially contribute to academic performance, controlling for the type of course (regular versus vocational), a hierarchical multiple linear regression model was tested. The hierarchical regression model was performed within four steps evaluating whether personality dimensions (2nd step), the type of learning approach (3rd step) and, the type of well-being (4th step), contribute to academic performance, controlling for the type of course (1st step). This model also evaluated how much additional variance of academic performance is explained by each of these variables/steps. All the scales of JTCI were included in the regression models regardless of their significant relationship with the dependent variable (academic performance). The premises for conducting Multiple Linear Regression were met, namely, linearity, homogeneity of variances and multicollinearity (such as Variance Inflation Factor-VIF) scores below 10 and tolerance scores above 0.2). Mediation analyses to test the role of learning approach as a mediator between personality and academic performance were carried out using the PROCESS macro for SPSS. All the analyses were performed with Software IBM^®^ SPSS^®^, version 25.0. A significant level of *p*-value ≤ 0.05 was assumed.

## 3. Results

### 3.1. Relationships between Personality, Learning Approaches, Affective and Non-Affective Well-Being, and Academic Performance

Table 1 shows the relationships between personality, learning approaches, affective and non-affective well-being, and academic performance.

### 3.2. Hierarchical Multiple Regression Testing Personality, Deep and Surface Approaches to Learning and Well-Being, as Predictors of Students’ Academic Performance

The hierarchical multiple regression model (Table 2), tested the variance of academic performance explained by personality dimensions, type of learning approach, and well-being, controlling for the type of course. The first step controlled for the type of course in which students were enrolled explaining 1% of the variance. The second step included personality dimensions and explained 11% of the variance of academic performance. The third step included learning approaches and the model explained 14% of the variance. The fourth step added affective and non-affective well-being and the model explained 15% of the variance of academic performance The final model explained 15% of the variance, F(12,580) = 8.477, *p* < 0.001. Personality dimensions added 10% of variance to the model, the type of learning approach added 3% and well-being added 1% of variance to the model.

### 3.3. Approach to Learning as a Mediator between Personality and Academic Performance

**Figure 1:**
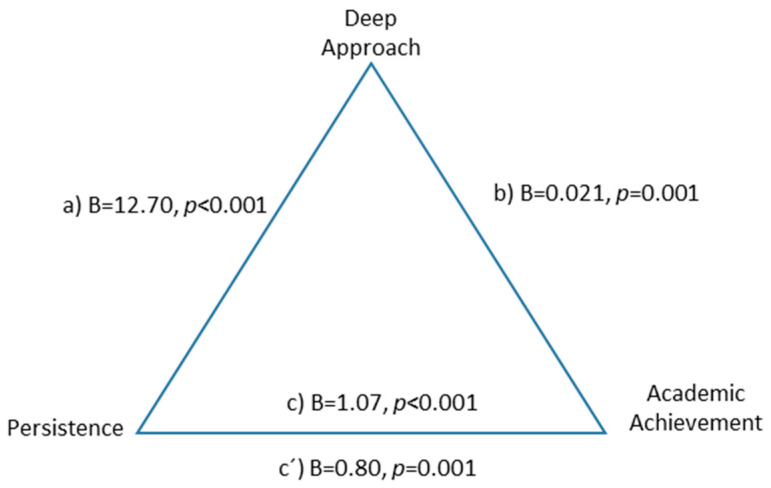
Partial mediation of deep approach to learning between persistence and academic performance.

Personality dimensions were associated with academic performance but, in order to increase knowledge about the mechanism by which they influence academic performance, a set of mediation analyses were carried out to explore the role of the type of learning approach as a mediator in this relationship. The deep learning approach proved to be a partial mediator in the relationship between persistence and academic performance, suggesting that the positive relationship between persistence and academic performance is partially mediated by the deep learning approach, F(2.590) = 23.36, *p* < 0.001, explaining 27% of the variance. The indirect effect was B = 0.270 BootSE = 0.077 (0.120 0.426) (Figure 1).

**Figure 2:**
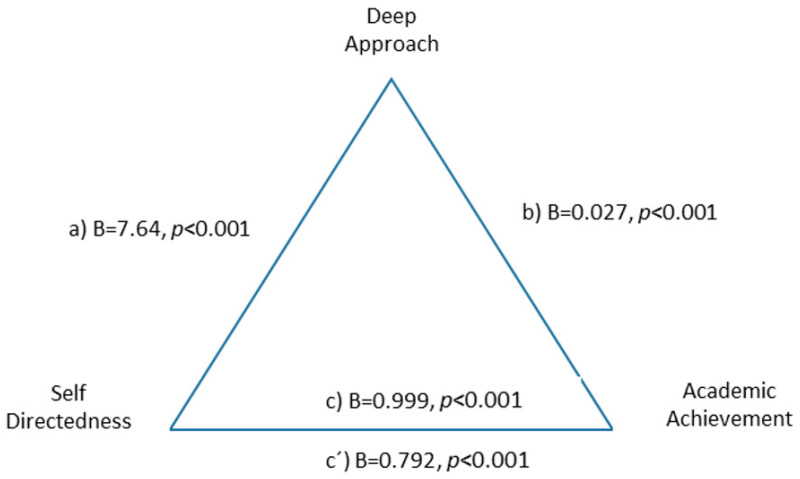
Partial mediation of deep approach to learning between self-directedness and academic performance.

The deep learning approach was a partial mediator in the relationship between self-directedness and academic performance, suggesting that the positive relationship between self-directedness and academic performance is partially mediated by the deep learning approach, F(2.590) = 21.90, *p* < 0.001, explaining 26% of the variance. The indirect effect was B = 0.206 BootSE = 0.059 (0.986 0.328) (Figure 2).

**Figure 3:**
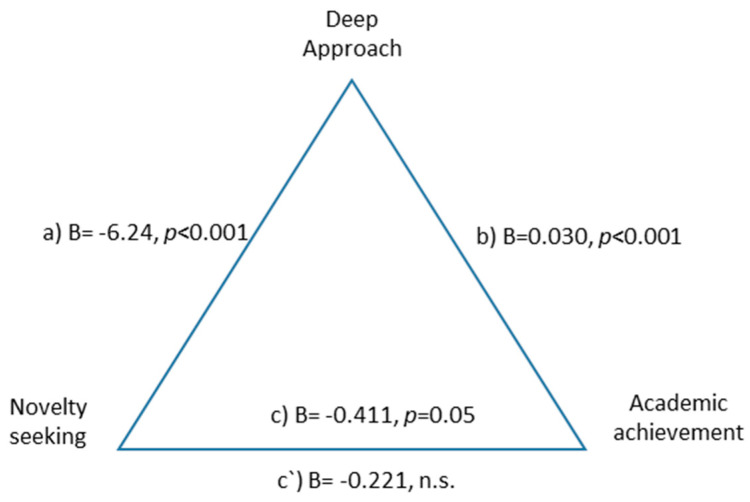
Total mediation of deep approach to learning between novelty seeking and academic performance.

The deep learning approach was a total mediator in the relationship between novelty seeking and academic performance, suggesting that the negative relationship between novelty seeking and academic performance is mediated by the deep learning approach, F(2.590) = 15.32, *p* < 0.001, explaining 22% of the variance. The indirect effect was B = −0.190 BootSE = 0.057 (−0.309 −0.091) (Figure 3).

**Figure 4:**
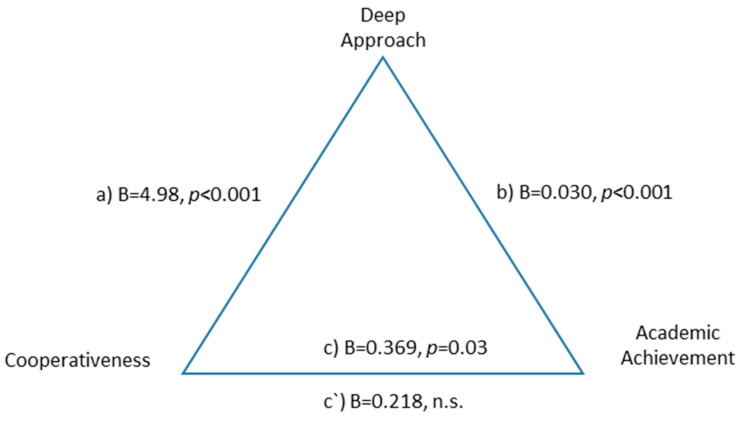
Total mediation of deep approach between cooperativeness and academic performance.

The deep learning approach was a total mediator in the relationship between cooperativeness and academic performance, suggesting that the positive relationship between cooperativeness and academic performance is mediated by the type of deep learning approach, F(2.590) = 15.57, *p* < 0.001, explaining 22% of the variance. The indirect effect was B = 0.151 BootSE = 0.049 (0.065 0.257) (Figure 4).

The surface approach to learning was not a mediator between personality and academic performance.

## 4. Discussion

This study aimed to understand the differential contribution of personality, the type of approach to learning, and the type of well-being for academic performance, as well as the mediating role of the type of approach to learning. The results showed that novelty seeking (high), harm avoidance (high), persistence (high), self-directedness (high), and self-transcendence (low) were the personality dimensions that contributed to academic performance [37,38,63,72]. The deep approach to learning has proven to be a significant predictor of academic performance and a significant mediator of the relationship between personality (novelty seeking, persistence, cooperativeness, and self-directedness) and academic performance. Affective well-being was a significant negative predictor of academic performance, unlike non-affective well-being, which was not a significant predictor. These results were significant controlling the type of course in which students were enrolled (students on vocational courses) and the variation explained in academic performance was residual (1%).

According to the Psychobiological Model of Personality [12], individuals high in novelty seeking are impulsive, curious, and enthusiastic, easily engaging in new ideas, activities, and tasks. For these individuals, everything is a challenge, and they are described as people who “hunger for knowledge” ([73], p. 842). Therefore, this is a personality trait that facilitates learning.

Individuals high in harm avoidance are pessimistic, fearful, worried, and frightened; they avoid novel stimuli and show a slow adaptation to new situations [12]. However, one of the advantages of high levels of harm avoidance is the greater care and caution with which they anticipate possible hazards, and carefully plan tasks and activities. As academic achievement is associated with a high inhibitory control (measured by high harm avoidance) [23], they may need more reinforcement and attention from teachers.

Individuals high in persistence are hardworking, persistent, stable, effortful, ambitious, responsible, and perfectionist workers, despite frustration and fatigue (which are perceived as a challenge). Besides, highly persistent individuals tend to set more challenging goals and commit to pre-defined tasks when compared to individuals with low levels of persistence. This personality trait is in accordance with the principles of a deep approach to learning, characterized by an intrinsic motivation to maximize intellectual understanding and extract meaning from the task [27,28,29,30].

Self-directedness refers to the individual’s ability to control and guide his conduct towards personal goals and objectives, using his resources appropriately [12,16]. Directional individuals are mature, strong, self-reliant, responsible, goal-oriented, constructive, effective, and able to adapt their behavior to personal choices and voluntary goals. Also, this dimension is associated with good self-esteem and a history of consistent bonding.

Individuals with low self-transcendence have low spirituality and little awareness of being part of a holistic reality that transcends their own individuality [12]. For this reason, students with low self-transcendence have a profile characterized by an organized and practical but not creative structure; they are materialistic, task-focused, and socially adapted, thus, achieving good academic performance.

Thus, individuals with high novelty seeking, harm avoidance, persistence, and self-directedness, but with low self-transcendence, showed better academic performance. The results are in line with previous studies [31,36,37,40,51]. Of the set of variables included in the model, personality was the one that explained the greatest variance in academic performance (11%), confirming hypothesis 1. The type of approach to learning and well-being explained a residual variance, emphasizing the role of personality in academic performance when approached in conjunction with the type of learning and well-being. Hypothesis 2 was not confirmed.

To explore the mechanism by which personality influence academic performance, we tested the role of learning approach as a mediator, and the results showed that a deep approach was a significant mediator of academic performance, as other studies have also highlighted, but not the surface approach [37,38]. Thus, our results add knowledge about the total mediation effect of deep approach in the relationship between persistence and self-directedness, and academic performance, which suggests that this type of approach to learning has a unique and independent positive effect on academic performance in addition to the explained effect by personality. The deep approach was a total mediator in the relationship between cooperativeness and novelty seeking, and academic performance. The results showed that the negative relationship between novelty seeking and academic performance no longer exists in the presence of a deep approach to learning, emphasizing the unique and independent positive effect of the deep approach on academic performance, in addition to the effect explained by cooperativeness. These results confirmed hypothesis 3 and are in accordance with the results of Yongjun and Reese’s [61] study.

Interestingly, affective well-being was a significant negative predictor of academic performance, while non-affective well-being was not a significant predictor, unlike the results found in other studies [50,74]. However, a recent meta-analysis of the relationship between academic performance and subjective well-being concluded that students with low performance do not necessarily report low well-being and that high-performance students do not automatically show high well-being [75], that is, a relatively small effect was found for the relationship of well-being and academic performance. The same was found in our study: academic performance and well-being were statistically significant but only relatively weak. We believe that students with higher academic performance are also more anxious, dedicated, and focused and, therefore, dedicate themselves more to studies, obtaining better grades, but sacrificing their well-being.

As hypothesized personality traits influence the relationship between well-being and academic performance. Novelty seeking is a personality trait that facilitates learning because it activates behavior [12,73], but harm avoidance inhibits behavior, and adolescents with high levels of this trait are pessimistic, fearful, concerned, and frightened [12]. As academic performance is associated with a high inhibitory control (measured by high harm avoidance) [31], these adolescents may need more reinforcement, attention and support from teachers. In addition, highly persistent adolescents tend to set more challenging goals, are hardworking, and, due to high levels of self-directedness, are more self-reliant, responsible, and constructive [12,16]. Finally, adolescents with low self-transcendence need to have control over everything: they are materialistic and very focused (even too much) on studies/work, seem dissatisfied with what they have in life, do not establish strong relationships with nature and people and, therefore, may have low well-being, as found in this study. Thus, hypothesis 4 was partially confirmed.

### 4.1. Limitations and Directions for Future Research

This study has some limitations that should be acknowledged, such as the cross-sectional design, which prevents us from establishing causal relationships, and the sample collection occurred in the North of the country, making it difficult to generalize the results to the whole country. Three alpha values found in this study are considered low (HA, RD, and PS dimensions). The use of measures to correct these alpha values was not considered because of the empirical validation that the model has been receiving both in different populations, ages, and functioning domains. On the one hand, the Psychobiological Model of Personality is a model that is characterized by having a complex factorial structure, which tends to have implications for the factor pattern matrix [76], often reflecting on the reliability of the scale. On the other hand, the same instrument presented higher and acceptable reliability values in previous studies in the current population [23,68]. However, and in spite of the robust empirical validation of the model, results of our study need to be considered with some caution, and future studies need to overcome this limitation.

Economic or social indicators, such as income or family size, were not controlled in this study and, therefore, future studies should control these variables. In addition, the variables addressed in this study focused exclusively on the student. Bearing in mind the importance of the discipline-teaching process interaction in determining the types of learning approaches, future studies should include variables that focus on students but also on the context [77].

### 4.2. Study Strengths

Although research describing the influences of learning and personality approaches on academic performance is abundant, studies on the interaction between personality (including temperament), learning approaches, and well-being in predicting academic performance, are scarce. Previous research has already shown consistent and positive associations between neuroticism and surface approach, and between conscientiousness and deep approach [19,20]. Recently, using Cloninger’s Psychobiological Model of Personality, Moreira et al. [23] found that, although students’ preferences for deep and surface learning approaches are best understood as integrated character and temperament profiles, temperament and character dimensions when analyzed separately, are associated with different learning approaches. Thus, this study adds to the existing literature and knowledge about the contribution of personality dimensions above and beyond the learning approaches and well-being, in a sample of high school adolescents.

### 4.3. Implications for Practice

Taking into account the variance in academic performance explained by personality, programs that nurture personality should be developed and implemented in schools. Despite the contribution of the deep approach to learning and the fragile relationship with affective well-being, academic performance seems to be more dependent on personality characteristics. Therefore, school-based policies and practices to promote academic performance should include activities and programs that promote a healthy personality development [78]. In addition, it is important to understand the students’ preferred approach to learning because the way students approach a learning task strongly influences the quality of learning outcomes [79]. Moreover, a deep approach to learning was a mediator between personality and academic performance, emphasizing that there are relationships that occur only in students who adopt a deep approach to learning. Besides the fact that personality predicts learning approaches [23], a recent study demonstrates that different combinations of temperament and character profiles significantly predict different dimensions of student engagement with school [80]. Together with these results, our study supports the need for schools to assume their responsibility in promoting students positive holistic development, by the systematic promotion of personality development and well-being [81].

Thus, well-being must be considered a fundamental educational asset in the conceptualization of education as a vital resource of society to face current and future challenges, such as sustainable development. A new paradigm in education is required for schools to be more efficient in preparing students to deal with the challenges that humanity faces, such as the need to promote sustainable behaviors [82,83]. “Personality development is a core dimension of holistic development and the most promising pathway for societies to promote youths holistic development is to move to person-centered schools” ([84], p. 183).

## 5. Conclusions

The results improve the understanding of the differential contribution of personality, type of learning approach, and type of well-being to academic performance. Understanding that personality is the strongest predictor of academic performance, after controlling the type of learning approach and the type of well-being, informs school policies and decision-makers that it is essential to encourage personality development in adolescents to improve academic performance.

## Figures and Tables

**Table 1:** Relationships between personality, type of learning approach, well-being, and academic performance (*n* = 593).

	1.	2.	3.	4.	5.	6.	7.	8.	9.	10.	11.	12.	13.	14.	15.
1. Academic Performance		0.074	0.022	−0.116 **	−0.082 *	0.078	0.063	0.235 **	0.190 **	0.088 *	−0.031	0.218 **	−0.037	0.081 *	−0.032
2. Gender			0.031	−0.049	−0.096 *	0.268 **	0.278 **	0.158 **	0.025	0.236 **	0.126 **	0.089 *	−0.145 **	−0.091 *	−0.092 *
3. Age				−0.326 **	0.027	0.050	0.002	−0.128 **	−0.077	−0.055	−0.006	−0.168 **	−0.194 **	−0.022	−0.042
4. Type of course					0.115 **	−0.027	0.036	−0.025	−0.046	−0.124 **	0.004	0.110 **	0.128 **	0.031	0.033
5. Novelty seeking						−0.570 **	−0.213 **	−0.454 **	−0.376 **	−0.437 **	−0.073	−0.176 **	0.179 **	−0.018	−0.131 **
6. Harm avoidance							0.019	−0.031	−0.367 **	0.035	0.009	−0.027	−0.064	−0.061	−0.258 **
7. Reward dependence								0.288 **	0.244 **	0.483 **	0.293 **	0.126 **	0.028	−0.034	0.199 **
8. Persistence									0.479 **	0.457 **	0.073	0.406 **	−0.003	−0.005	0.190 **
9. Self-directedness										0.427 **	0.107 **	0.210 **	−0.105 **	−0.039	0.319 **
10. Cooperativeness											0.296 **	0.166 **	−0.093 *	−0.060	0.119 **
11. Self-transcendence												0.164 **	−0.032	−0.050	−0.026
12. Deep approach													0.233 **	0.198 **	0.109 **
13. Surface approach														0.131 **	0.021
14. Non-affective well-being															−0.011
15. Affective well-being															

Note. Type of course and gender were coded as a dummy variable with vocational courses = 0 and regular courses = 1; boys = 0 and girls = 1; ** *p* < 0.01, * *p* < 0.05.

**Table 2:** The summary output of hierarchical multiple regression model testing personality, type of approach to learning, and well-being as predictors of students’ academic performance.

		*R* ^2^	*R* ^2^ *_adj_*	*F*	*β*	*p*
Step 1						
Course type		0.013	0.012	7.991 **	−0.116	0.005
Step 2		0.108	0.096	8.854 ***		
Course type					−0.115	0.004
Novelty seeking					0.112	0.021
Harm avoidance					0.189	<0.001
Reward dependence					0.021	0.644
Persistence					0.213	<0.001
Self−directedness					0.227	<0.001
Cooperativeness					−0.076	0.152
Self-transcendence					−0.047	0.263
Δ R^2^	0.095 ***					
Step 3		0.136	0.121	90.149 ***		
Course type					−0.131	0.001
Novelty seeking					0.126	0.009
Harm avoidance					0.186	<0.001
Reward dependence					0.030	0.510
Persistence					0.141	0.006
Self-directedness					0.215	<0.001
Cooperativeness					−0.067	0.203
Self-transcendence					−0.079	0.060
Deep approach					0.192	<0.001
Surface approach					−0.059	0.156
Δ R^2^	0.028 ***					
Step 4		0.149	0.132	8.477 ***		
Course type					−0.127	0.001
Novelty seeking					0.127	0.009
Harm avoidance					0.176	<0.001
Reward dependence					0.047	0.304
Persistence					0.151	0.003
Self-directedness					0.242	<0.001
Cooperativeness					−0.071	0.174
Self-transcendence					−0.083	0.048
Deep approach					0.179	<0.001
Surface approach					−0.063	0.125
Non-affective well-being					0.077	0.054
Affective well-being					−0.095	0.024
Δ R^2^	0.013 *					

Note. Course type was coded as a dummy variable with vocational courses = 0 and regular courses = 1; *** *p* < 0.001, ** *p* < 0.01, * *p* < 0.05.
